# How does GP training impact rural and remote underserved communities? Exploring community and professional perceptions

**DOI:** 10.1186/s12913-020-05684-7

**Published:** 2020-08-31

**Authors:** Katerina Kanakis, Louise Young, Carole Reeve, Richard Hays, Tarun Sen Gupta, Bunmi Malau-Aduli

**Affiliations:** James Cook University, Townsville, QLD 4814 Australia

**Keywords:** GP training, Underserved communities, Rural, Remote, Family medicine, Impact, Socio-economic

## Abstract

**Background:**

Substantial government funding has been invested to support the training of General Practitioners (GPs) in Australia to serve rural communities. However, there is little data on the impact of this expanded training on smaller communities, particularly for smaller rural and more remote communities. Improved understanding of the impact of training on underserved communities will assist in addressing this gap and inform ongoing investment by governments and communities.

**Method:**

A purposive sample of GP supervisors, GP registrars, practice managers and health services staff, and community members (*n* = 40) from previously identified areas of workforce need in rural and remote North-West Queensland were recruited for this qualitative study. Participants had lived in their communities for periods ranging from a few months to 63 years (Median = 12 years). Semi-structured interviews and a focus group were conducted to explore how establishing GP training placements impacts underserved communities from a health workforce, health outcomes, economic and social perspective. The data were then analysed using thematic analysis.

**Results:**

Participants reported they perceived GP training to improve communities’ health services and health status (accessibility, continuity of care, GP workforce, health status, quality of health care and sustainable health care), some social factors (community connectedness and relationships), cultural factors (values and identity), financial factors (economy and employment) and education (rural pathway). Further, benefits to the registrars (breadth of training, community-specific knowledge, quality of training, and relationships with the community) were reported that also contributed to community development.

**Conclusion:**

GP training and supervision is possible in smaller and more remote underserved communities and is perceived positively. Training GP registrars in smaller, more remote communities, matches their training more closely with the comprehensive primary care services needed by these communities.

## Background

Substantial government funding has been invested over the last 20 years to expand training for the specialty of general practice (GP) with the aim of improving the medical workforce, particularly outside of larger urban centres. During this time period, the numbers of medical graduates has increased three-fold and the number of graduates with rural placement experience and rural career interest have risen [1]. However, the numbers working in rural and remote communities after specialist training has not increased as much, particularly in smaller and more remote communities [2]. Many more graduates now train and remain in larger regional and rural communities, where there is capacity for both clinical workload and supervision by experienced GP supervisors. In smaller, more remote communities, training opportunities have been constrained by the scarcity of learning resources, particularly experienced GP supervisors, who may be overloaded with clinical service. The question has been posed: would placing GP registrars in such communities be a drain on existing, insufficient resources or a mechanism for investment in the sustainability of health services for otherwise underserved communities?

Expanding training in smaller centres requires a distributed model that places resources, including clinical facilities, IT infrastructure and appropriate supervision, in practices and communities that could not otherwise support training. While most evaluations of medical workforce initiatives have focused on numbers of doctor, the potential impacts are much broader, including community and economic outcomes [3–5]. An improved understanding of the impact of training on underserved communities would address this gap, guide further development of the training program to maximise Government investment, and identify the benefits of additional health services in the community.

### Health care in smaller and more remote communities

Australians living in rural and remote areas experience lower levels of health and life expectancy when compared with the population in metropolitan areas [6]. They are less likely to have a nearby or regular GP and more likely to present to an emergency department [7]. They are also more likely to report poor access to adequate health care [8]. Greater distance to major centres is a major factor, with residents having to travel, sometimes for substantial distances, with associated increases in costs and disruptions to family, employment and broader community activities [9, 10]. Smaller local populations also reduce the range of resident (full-time) medical services available, so narrower specialty services are even less available and accessible.

The composition of the local medical workforce may influence local community perception. Increasingly, medical services are not provided solely by local resident doctors who are part of the community. Sustaining this model is difficult so the workforce is supported by either fly-in, fly-out’ (FIFO) or locum workers. The former can be more stable if the same doctors come in regularly, however the latter may be more disruptive. There is some evidence that a less stable medical workforce is less popular with smaller communities [8, 11–13]. Continuity of care is more difficult [14] and trusting relationships with doctors are less likely [15] due to constant turnover.

### Developing a medical workforce ‘pathway’

Undergraduate initiatives that promote rural career interest through rural placements and role models are the beginning of the pathway. There is increasing evidence that such a pathway can attract applicants from an increasing pool of medical graduates with substantial rural experience and interest. Socially accountable medical schools, including James Cook University (JCU), produce a substantial proportion of medical graduates interested in working in underserved areas [16–18]. These graduates are also often more ‘work-ready’ at graduation due to their extensive (a minimum of 20 weeks rural placement), community-engaged clinical exposure in underserved communities [19–34].

The final component of training for rural practice is four years of postgraduate training and assessment that leads to a Fellowship of either the Royal Australian College of General Practitioners (RACGP) or the Australian College of Rural and Remote Medicine (ACRRM) and a licence for independent practice [35, 36]. The gap between undergraduate and postgraduate training has long been a problem, with graduates responsible for gaining pre-registration experiential training in hospitals and then competing in an open selection process for postgraduate training places. For many, this middle period is full of anxiety and uncertainty. In response, a rural pathway has been established to align longitudinally with undergraduate and postgraduate initiatives. Students who participate in undergraduate rural initiatives may achieve preference for postgraduate rural training places [2]. A well-known rural pathway is the Queensland Rural Generalist Program (QRGP), which attracts, trains and supports a medical workforce for smaller rural hospitals [17, 37].

JCU has developed a local version of the rural pathway, integrating JCU’s postgraduate GP Training Program (JCU GPT) with the successful distributed undergraduate program, and working closely with the QRGP to place GP registrars with an interest in rural medicine in smaller, more remote communities. Training is localised through the devolution of training to 10 hubs, each with local offices to support rural doctor training within the context where doctors are most needed. This intervention is referred to as “localised GP training” in this article. The financial investment was substantial: direct expenditure for 2016–2018 by JCU was over $49 million, including administration, office space, travel, support for supervisors, practices, registrars and and local medical educators ($33,845,520), and salary costs ($15,266.424). According to the approach used by Hogenbirk et al. in Northern Ontario [3], where direct financial investment by the training provider is modified by population-based multipliers to account for re-spending within the community, this suggests a total of an additional $90 million dollars circulating in the communities hosting localised GP training.

A feature of the JCU rural pathway is strong engagement and reciprocal relationships with the selected communities and the employers of the GP Registrars [38], a strategy that should be a starting point for rural community-based medical education [39]. These specific relationships included personal-professional, clinician-patient, university-health service and government-community. For communities in particular, contributing to the selection of doctors, health governance, teaching, and welcoming and supporting doctors and students during community placements are essential for symbiotic medical education to occur [17]. Symbiotic relationships may increase patient centred care [40] and workforce recruitment [16]. These features make even more important the need for research that considers social and community, as well as health and economic, outcomes of the expanded localised GP training approach.

**Fig. 1:**
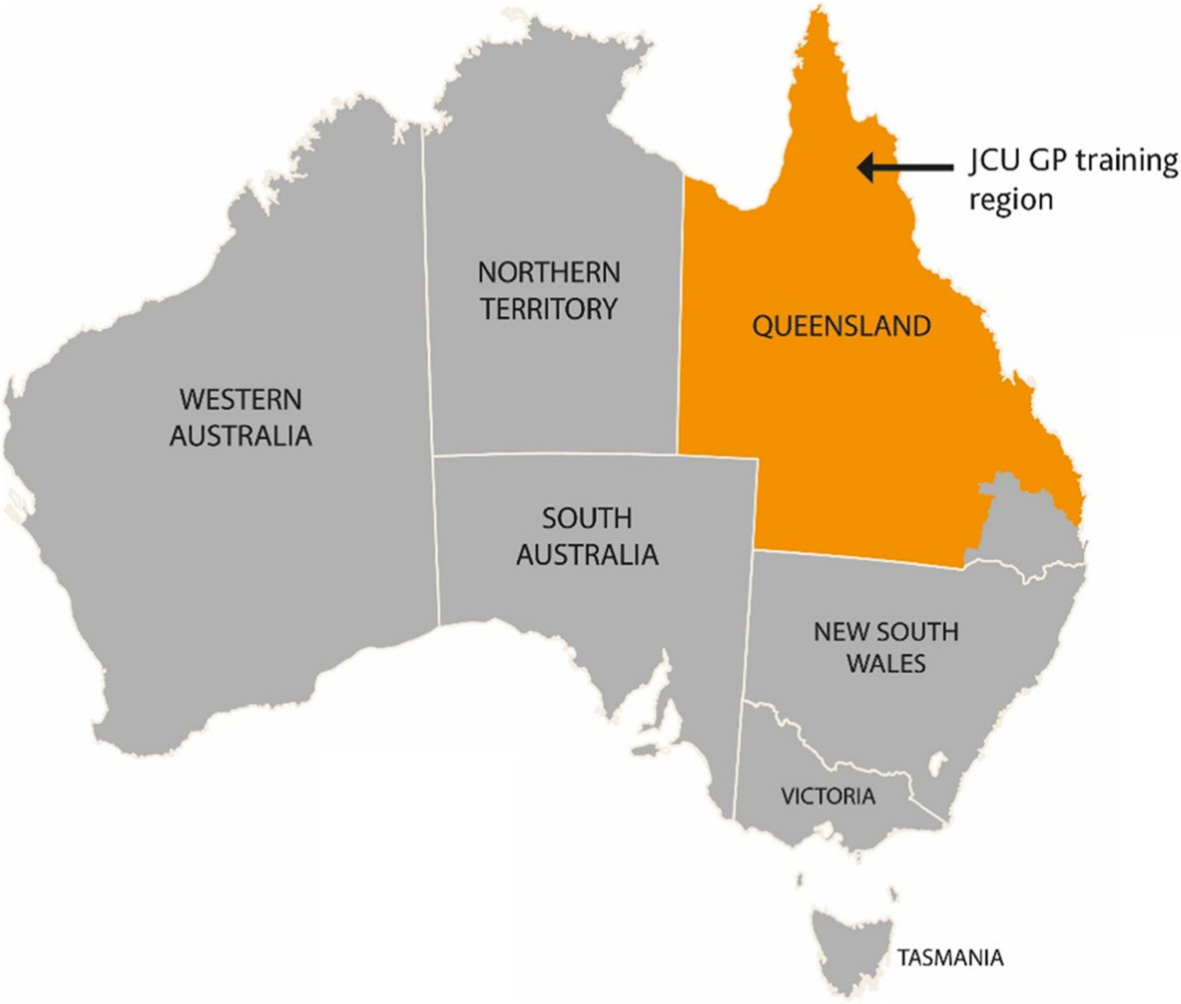
James Cook University GP Training area (image provided by JCU GPT)

The aim of this study was to explore the workforce, health service and social impacts on communities arising from the expansion of the JCU GPT program into smaller, underserved rural and remote communities. Changes in health status and economic outcomes for communities as a result of GP training were beyond the scope of this study. Measuring economic impact beyond the input is difficult. An exploratory, qualitative approach was taken to gain a deeper understanding of the impact of training on these communities. Since 2016, JCU GPT, as one of nine GP training providers in Australia, has provided GP training for over 90% of the geographically large state of Queensland, including many regional, rural, and remote areas, as shown in Fig. 1 [17, 41]. While definitions and uses of the term ‘community’ vary in the literature [42], we have adopted a geographic definition relevant to rural and remote Queensland, where relatively small populations face a degree of physical isolation and residents share the same services, local government and a sense of belonging [43].

## Method

### Participants

A purposive sample of GP supervisors, GP registrars, practice managers and health services staff, and general community members from Queensland communities with low medical workforce were recruited for this study [44]. The Modified Monash Model (MMM) remoteness classification for the targeted communities was 4–7, ie rural communities of less than 15,000 and remote locations. This classification is based on Australian Bureau of Statistics distance and population data, ranging from one (major cities) to seven (very remote) [45]. Invitations to participate were sent by email through local networks to key informants in the underserved communities (such as training staff), relevant federal, state and local level government members, healthcare services and groups, and community service groups (such as Rotary, Country Women’s Association). Prospective participants were eligible to participate as long as they were residents of the targeted communities and had some knowledge of the training program. No limit was placed on the number of participants required as strict guidelines regarding sample sizes are not used in qualitative research due to the importance that is placed upon the individual’s experience [46]. A total of 40 participants participated in the project. Participants had lived in their communities ranging from a couple of months to 63 years (Median = 12 years). See Table 1 for demographic details.

**Table 1:** Participant Demographics

Characteristics		Total
Gender	Female	19
	Male	21
Age	20–30	9
	31–40	2
	41–50	7
	51–60	8
	61–70	9
	71–80	5
Group	Community Member	21
	Supervisor	7
	Registrar	8
	Practice Manager and Healthcare Services Staff	4
Region (MMM)	Cape York (6)	3
	Central Queensland (4)	7
	Central West Queensland (7)	17
	North West Queensland (6)	7
	South West Queensland (4–6)	3
	Wide Bay (5)	3

### Procedure

Semi-structured interviews and a focus group were conducted to explore how establishing GP training sites impacts underserved communities from a health workforce (for example, what has been the impact of having JCU GPT doctors training in your community on the doctors or other healthcare providers in your community?), health outcomes (for example, what has been the impact or result of having GP training locally on the availability of healthcare services?), economic (for example, have there been any examples you can think of where the JCU GP training program has contributed to the local economy?), and social perspective (for example, how have the JCU GPT doctors or their families contributed to the community outside of work?”. Interview questions were used as a guide and prompt for information and were not prescriptive. Interview guides were developed by the research team based on previous literature and the identified gaps in the literature relating to the impact of training on rural and remote communities. This article formed part of a broader project and therefore not all questions within the interviews were relevant to the findings presented within the article. Interviews were conducted from November 2018 to March 2019, either over the phone or face-to-face at a time and place chosen by the participant. The focus group was held with six registrars during a training day for ease of participation. Interviews and the focus group were audio-recorded and transcribed verbatim and lasted between 20 min to one hour. Interviews were analysed as they were completed to identify when data saturation had been reached and no further recruitment was necessary. No new major themes were identified after the 30th interview. Recruitment and interviews continued until data saturation was reached [47]. One researcher (KK) who conducted the interviews and focus group was employed as a research officer for JCU GPT and did not know the participants prior to the interviews and focus group. This researcher had experience working as a qualitative research officer in other projects and had conducted a PhD using qualitative methods. Another researcher (CR) was involved in conducting some interviews and was employed by JCU GPT. One additional researcher (RH) was involved in conducting the focus group and was a medical educator employed by JCU.

### Analysis

Interview data was analysed using thematic analysis which provides a systematic process to identify, analyse and report on patterns within data. Analysis was conducted as an iterative process of six steps (data familiarisation, general coding, organisation, revision, and definition of themes, analysis report) as described by Braun and Clarke [48]. Analysis occurred while interviews were still being conducted. Initial codes were generated through manifest-content analysis where the visible and/or apparent content of the transcripts were coded [49]. Emerging themes were confirmed using shared coding sessions and theme generation by two researchers (KK and LY) where consensus was used to resolve any discrepancies. In addition to the research officer who collected the data (KK), analysis was also conducted by a researcher (LY) who is a medical educator with JCU. All authors confirmed the final identified themes. Data analysis was aided by the use of the qualitative data software NVivo 12 [50].

## Results

Interview data indicated a positive impact of localised GP training on communities as reported by participants. For example, participants reported that, *“GP training has all worked very well. And we don’t want it to go away”* (Community 2), and that, *“the positives would far outweigh [the negatives]”* (Community 16). Participants’ reports of the impact of localised GP training reflected some important themes which are described below: health status and services, social factors, financial factors, cultural factors and education.

### Health status and services

The most commonly reported impacts on the community resulting from the GP training program concerned health status and services and included both positive and negative impacts. Participants’ reports of their community’s health status and services related to comments about quality of health care, accessibility of health care, continuity of care, the GP workforce, health status, and preventative health care. Overall, participants reported positive perceptions of the impact that GP training had on the health status and provision of health services in the community. Table 2 presents these positive perceptions and further description of the themes. Negative responses referred to either no perceived change in health status or services, or a desire for more of the positive changes that had been occurring as a result of the training program. For example, participants reported that, *“I don’t think it’s changed the availability of healthcare services and such”* (Supervisor 3) and also that, “*… I think we’ve got one registrar and one Fellowed doctor … So we probably need to see more before we can say that there is definitely a positive impact”* (Supervisor 1).

**Table 2:** Health Status and Services

Theme	Description	Example
Quality of Health care	Health service quality, professional relationships between registrars and patients, rural competency and new approaches	“*… having registrars improved the quality.”* (Supervisor 7) “*… just for being in the community and knowing the community, will start some confidence and therefore has a better understanding and is going to be able to deliver care with a better understanding.”* (Supervisor 2) *“I think it’s always good, particularly in these smaller rural areas where younger people with new ideas bring those into practices. That has seemed to have been a benefit to the outcome of the program … some fresh ideas.”* (Community 21)
Accessibility	Access to doctors and services, decreased travel and decreased waiting times	“*… by having the extra training, we’ve been able to get an increased access to doctors, so that’s very good.”* (Community 6) “*… [practices] are able to offer a service that they might not have been able to without [the registrars].”* (Community 21) “*… patients stop travelling for their primary care.”* (Community 4) “*… the waiting time is down to bugger all.”* (Community 9)
Continuity of Care	Patient continuity of care	“*… because we’re a training practice … it means that we do have doctors that are here for a year or two, even if they’re just registrars, providing good continuity of care.”* (Supervisor 3) “*… the continuity of care after that teleconference … is where the registrars … come in really strongly.”* (Health Staff 3)
GP Workforce	Recruitment and retention of GPs and sustainability of health care practices and practitioners	“*… the effect of having access to training for registrars in rural and remote communities has significantly influenced the ability to recruit and retain a medical workforce.”* (Community 4) *“[The localised GP training program]‘s been a revelation, to be honest with you and it’s stabilising the workforce.”* (Community 9)
Health Status	Community health outcomes	*“we’re seeing … a lot better health outcomes for patients”* (Community 8) *“But it’s also encouraging the community, particularly the outlying communities, to take more responsibility for their health.”* (Community 9)
Preventative Health care	Health promotion and preventative healthcare activities	“*… preventative health care … was definitely something that could not have been done without registrars.”* (Supervisor 7) “*… the interns last year attended our local show and ran a stall with one of the private practices here for a bit of people’s health scans … I think that that’s a hell of a good exposure … to be able to promote rural medicine and, as well as that, just provide … some good, healthy, outcomes.”* (Community 8)

### Financial factors

Participants reported financial benefits for their community including general spending, employment, housing and infrastructure. Overall, participants reported a positive influence on financial factors in the community as a result of the training program or the registrars involved. Table 3 presents these positive perceptions and further description of the themes. Negative responses noted no perceived change to community financial factors. For example, one participant commented that the localised GP training program did not impact the community “*… in a big way, no, because there’s not that many [registrars]”* (Supervisor 1).

**Table 3:** Financial Factors Themes

Theme	Description	Example
General Spending	Economic contribution from spending in community	*“The doctors come out, they spend their money and like everyone else, they just put their money through the town and it bolsters things.”* (Community 12) “*… they buy their groceries, they pay their rent or use whatever local businesses they can. So obviously the more people you can attract to a small community, it affects the economy of the town.”* (Community 3)
Employment	Employment due to training	“*… the staff numbers have increased.”* (Community 4) “*… there are a certain number of people that have gained employment, whether it be full-time or part-time.”* (Community 9)
Housing	Housing for registrars	*“There’s extra housing available.”* (Community 4) “*… I know they rent homes for them which is a blessing for people that own homes that can’t be, occupied.”* (Community 11)
Infrastructure	Health care infrastructure	“*… there’s construction work being done to extend the practice. There’s rooms being rented by visiting services.”* (Community 4) “*… we’ve got the [university] campus in town, and that’s used for nursing training and other things as well.”* (Supervisor 5)

### Social factors

Participants’ reports about their community’s social factors included community participation and relationships. Participants reported an overall positive influence from the training program and registrars’ impact on the social aspects of their communities. Table 4 presents these positive perceptions and further description of the themes. Negative responses referred to a perceived lack of social contribution attributed to a perception of registrars being time poor. For example, one participant noted that “*… there’s not a lot of capacity for them to volunteer in the community, because they’re so busy and they’re still studying”* (Community 6).

**Table 4:** Social Factors Themes

Theme	Description	Example
Community Participation	Registrars’ participation within the community outside of work	“*… I do hear anecdotally that the doctors are out and about and they attend the local hotels and community events.”* (Community 20) “*… from our point of view, it’s been really good and you meet quite a lot of them. The thing I like about it is that they get involved in the community.”* (Community 2)
Relationships	Relationship between the community and healthcare services	“*… it’s probably the best sort of relationship between the community and the, hospital there has been … they tend to participate more in the community so you’re actually meeting them in other social aspects and things like that that you might not have before.”* (Community 13) *“There’s a good relationship, I must say … the trust in the hospital is really good.”* (Community 20)

### Cultural factors

Participants’ reports about their community’s cultural factors identified values and identity of rurality and remoteness. Participants reported only positive perceptions in terms of an appropriate fit between registrars and community identity. Participants stated that “*… our own trained doctors are much more desirable and a better fit … they know our culture, they know who we are”* (Community 9), and that “*… the community has confidence that this person knows what they’re talking about and is one of them”* (Supervisor 2).

### Educational factors

Participants described educational benefits to their community from the rural pathway and symbiotic relationships. Overall, participants reported a positive influence of the localised GP training program on their communities’ educational factors. Table 5 presents these positive perceptions and further description of the themes. One participant reported a negative perception regarding no perceived change to community educational factors when stating that, “*… I don’t think it’s really had an impact. We haven’t seen very many students … be remotely interested in medicine”* (Supervisor 1).

**Table 5:** Educational Factors Themes

Theme	Description	Example
Rural Pathway	Medicine perceived as a viable option for rural communities	*“Having that visibility of [the university] in the community shows people that there is another option for them … they don’t have to go to Brisbane … because if they go to Brisbane, they often never come back.”* (Community 21) *“I think that there are more and more local people, even … students from our high school that have been looking into medicine.”* (Community 9)
Symbiotic Relationships	Registrars learn from the community	*“I think [the registrars] probably learn a lot from people out here because … most people sort of say it as it is and they won’t beat around the bush with them … And I think they’ll benefit from that sort of interaction.”* (Community 12) “*… two things, to let us understand what they’re all about and then to get an understanding of what a rural community is and how it functions.”* (Community 20)

### Registrar factors

Participants reported benefits to the registrars within the program which also contributed to the community. Table 6 provides further description and examples of the themes. Scope of practice, increased registrar knowledge and breadth of training provided a perceived wider range and quality of health services for these communities.

**Table 6:** Registrar Themes

Theme	Description	Example
Breadth of Training	Breadth of training opportunities results in a wider range of services for communities	*“In actual fact … by using something like the Rural Generalist doctors program, where some doctors will get a specialty which they can use in the region - and whether that’s obstetrics or whether it’s anaesthetics or surgery, whether it’s mental health”* (Community 8) *“So I think one of the advantages of working in a really remote area is we just get to do such a wide variety that you wouldn’t necessarily get to do in a suburban practice.”* (Registrar 2)
Rural Specific Knowledge	Knowledge and understanding of rural areas provides a greater service	*“So we know that they … will have a much better understanding of what this community is about in terms of its type of industry, its type of specific problems, in what resources are available or not available.”* (Supervisor 2) *“So each area, by virtue of its isolation and the make-up of its community, will have different challenges. But what we are finding with these young folks coming through is that they really seem to have some knowledge and understanding of this. It makes a massive difference.”* (Health Staff 3)
Quality of Training	Quality of training provides registrars with greater level of skills and knowledge	*“The training program by itself has built a level of skillsets and knowledge that the GP is better able to then work with a local regional community than other GPs who haven’t gone through this.”* (Supervisor 2) “*… the registrar training I found has been extraordinarily effective.”* (Health Staff 3)
Relationships with Community	Registrars’ relationships with community influence community outcomes	*“Registrars become invested in local life and committed to patients.”* (Health Staff 1) “*… they’ve participated in activities and they’ve helped out at … functions, they’ve gone for bike rides with our riding group and things like that. So … there’s obviously a positive community, effect because they’re ultimately here in the community and they’re participating in things.”* (Community 13)

## Discussion

The aim of this study was to explore the workforce, health service, and social impacts on communities arising from the expansion of the JCU GPT program into smaller underserved rural and remote communities. The study results indicate that the training program’s approach of community-based medical education fosters positive health outcomes as well as social and financial benefits for these underserved regions and enhances registrars’ development of extensive clinical skills and community connectedness building on the benefits from rural undergraduate medical training [16–21, 23, 30, 31].

These results support and expand on previous undergraduate medical research [2, 32, 33] as participants reported a perceived improvement to health outcomes and financial benefits resulting from localised GP training. In particular, participants reported increased access to doctors and healthcare services as a result of the localised GP training in their community. This finding suggests that localised GP training may address many of the issues facing the health of rural and remote communities such as inability to access GPs or adequate health care and the requirement to travel for health care [4–7]. Additionally, participants reported a perceived increase in the quality and continuity of health care resulting from more registrars working in the community, rather than an ever changing supply of locums. This finding provides support for a shift away from a locum model towards providing localised GP training to increase the rural medical workforce. Therefore, there is a perceived improvement, not only in overall health status and outcomes, but also specifically to accessibility, continuity and quality of health care, including preventive healthcare services, and the availability and sustainability of the rural GP workforce.

In addition to the health outcomes and financial benefits, there were perceived improvements in the social, cultural and educational factors of the communities such as increased community group membership and participation in community events. These results highlight the wide ranging impacts that localised GP training may have on rural and remote communities, particularly when linked to a rural training pathway. The results further highlight there are perceived symbiotic relationships between the community and the registrars. Community members enjoy participating within training and providing guidance to registrars, thereby giving them a sense of ownership over their health [35]. This finding in particular suggests that not only may the community benefit from localised GP training, but that the registrars may also benefit from being exposed to the local community.

Participants also reported that localised GP training promoted rural medicine as a career within their community. This finding provides support for the idea that localised GP training may improve the rural pathway through promoting rural medicine within local schools. Similarly, participants noted that localised GP training played an important role in the recruitment and retention of GPs. In this way, the localised GP training program may contribute to promoting rural health professions as career pathways in multiple ways (e.g. create interest during school years and exposure to rural practice).

### Limitations

The current study explored the localised GP training program of only one training organisation in one state and therefore, the transferability of the results to other settings may be limited.. Repetition in other training settings could strengthen the dependability of these results Future studies could explore further the economic benefits of investment in localised training.

## Conclusion

Localised GP training can provide many benefits for rural and remote communities. The communities’ health, financial, social, cultural and educational factors are all perceived to be improved overall. Training GP registrars in smaller, more remote communities, seems to better match the scope of practice during training with that required for the provision of comprehensive primary care services needed by these underserved communities. Furthermore, registrars are provided with opportunities to build community connectedness and establish symbiotic relationships with communities.

## Supplementary information

**Additional file 1.** Focus Group Guide (Registrars).

**Additional file 2.** Interview Guide (Community Stakeholders).

**Additional file 3.** Interview Guide (Practice Managers/ Health Service Managers).

**Additional file 4.** Interview Guide (Registrars).

**Additional file 5.** Interview Guide (GP supervisors).

**Additional file 6.**

**Additional file 7.**

## Data Availability

The interview transcripts generated and analysed during the current study are not publicly available due to confidentiality of the participants but are available from the corresponding author on reasonable request.

## Abbreviations

ACRRM: Australian College of Rural and Remote Medicine
GP: General Practice/Practitioner
JCU: James Cook University
JCU GPT: James Cook University General Practice Training
MMM: Modified Monash Model
RACGP: Royal Australian College of General Practitioners
