# Supplementary material for: How does GP training impact rural and remote underserved communities? Exploring community and professional perceptions
Source: BMC Health Serv Res. 2020 Aug 31;20:812. doi: 10.1186/s12913-020-05684-7 (PMC7457499; doi:10.1186/s12913-020-05684-7)
Supplement: Supplementary file 1 — Additional file 1. Focus Group Guide (Registrars). [file 12913_2020_5684_MOESM1_ESM.docx]

**Focus Group Guide (Registrars)**

Date of the Interview: ____/____/____

The interview opens with:

*Hello, my name is* ***XXX****. I will be interviewing you today. The purpose of this interview is to understand the impact of GP registrar training in rural and remote areas. I will be audio recording this interview, which will be de-identified and remain confidential. Taking part in this study is voluntary and you can stop at any time without explanation or prejudice. Do you consent to participate in this focus group?*

*Thank you for consenting to participate in this focus group!*

*Before we start, I would like to know a bit about you.*

**Demographic Questions**

1. Do you have a partner or children and how has that influenced your decision to train remotely?

*Thank you very much for answering this question.*

*Now I will ask you a few questions related to practice and life in a rural/remote community*

**Quality of Medical Practice Training**

1. Did you choose the town/ practice for training and if so why?
2. What attracted you to rural and remote practice?

**SUPERVISION**

1. What kind of supervision do you most value in a clinical placement? *Can you please elaborate with examples?*
2. How would you describe your current clinical supervision?

**SOCIAL**

1. How do you contribute to the local community outside of work?
2. Can you tell give us examples of what you have learnt or experienced as a GP in this community that has enriched you at a professional or personal level?
3. How have you contributed most to health services in this community?

*If time permits:*

1. If you have chosen one or more advanced skills as a form of specialisation in a particular area of practice, which one did you choose?

b. Why did you choose that area?
