# Supplementary material for: How does GP training impact rural and remote underserved communities? Exploring community and professional perceptions
Source: BMC Health Serv Res. 2020 Aug 31;20:812. doi: 10.1186/s12913-020-05684-7 (PMC7457499; doi:10.1186/s12913-020-05684-7)
Supplement: Supplementary file 4 — Additional file 4. Interview Guide (Registrars). [file 12913_2020_5684_MOESM4_ESM.docx]

**Interview Guide (Registrars)**

Participant ID: __________________________

Community Location: ____________________

Date of the Interview: ____/____/____

Interview Mode:

In Person

Over the Phone

Key Informants’ Gender:

Male

Female

The interview opens with:

*Hello, my name is* ***XXX****. I will be interviewing you today. The purpose of this interview is to understand the impact of JCU GP training registrars in rural and remote areas. JCU has been providing GP training across North West Queensland for the past 3 years building on the undergraduate medical training program and we are trying to better understand the effects of the program on registrars and communities. I will be audio recording this interview, which will be de-identified and remain confidential. Taking part in this study is voluntary and you can stop at any time without explanation or prejudice. Do you consent to participate in this interview?*

After consent is given, proceed to ask some demographic questions.

*Thank you for consenting to participate in this interview!*

*Before we start, I would like to know a bit about you.*

**Demographic Questions**

1. Could you please tell me your age range?

20 – 30

31 – 40

41 – 50

51 – 60

61 – 70

71 – 80

Prefer not to say

2. Do you have a partner or children and how has that influenced your decision to train remotely?

3. How long have you lived/ practiced in this community (in years)?

*Thank you very much for answering these questions.*

*Now I will ask you a few questions related to practice and life in a rural/remote community*

**Quality of Medical Practice Training**

1. Why did you choose this town/ this practice for GP training?
2. a. How does your average day looks like in terms of the type of clinical work you do?

b. What is your scope of practice?

c. How are you sharing the care of patients with specialists? *Please give examples*

1. Thinking of the past year, excluding on-call, how many hours did you undertake work in each of these settings in a usual week at work.

Where?

1. Eg General practice- office based
2. Hospital based work eg ward work
3. Medical administration (examples rosters, meeting representative)

Specialty areas

1. Emergency department work
2. Obstetrics and deliveries
3. Anaesthetics
4. Palliative care
5. Population health/ preventive health/ health promotion

Other

1. Nursing homes/ aged care
2. Community work eg AMS, RFDS
3. Outreach
4. Telehealth
5. Teaching or supervision
6. Thinking of the past year, in a usual week at work:
7. How many hours were you rostered for after-hours/on call
8. How many hours (on call) did you actually spend in direct patient care
9. How many times (on call) were you called out
10. What percentage of your on-call shifts would have more than one call out?
11. If you have chosen one or more advanced skills as a form of specialisation in a particular area of practice, which one did you choose?

b. Why did you choose that area?

**SUPERVISION**

1. What kind of supervision do you expect in a clinical placement? *Can you please elaborate with examples?*
2. How would you describe your current clinical supervision?

**BACKGROUND**

1. Where did you grow up? Include name of pace Remote, Rural or Urban if unclear?

*Remote:* What attracted you to continue staying and practising in a rural area?

*Rural:* What attracted you to continue staying and practising in a rural area?

*Urban:* What was the transformative experience or defining moment for you to decide to practice in a rural area?

**SOCIAL**

1. How do you participate or contribute in the local community outside of work hours?
2. Can you give us examples of what you have learnt or experienced as a GP in this community that has enriched you on a professional or personal level?
