# Supplementary material for: How does GP training impact rural and remote underserved communities? Exploring community and professional perceptions
Source: BMC Health Serv Res. 2020 Aug 31;20:812. doi: 10.1186/s12913-020-05684-7 (PMC7457499; doi:10.1186/s12913-020-05684-7)
Supplement: Supplementary file 5 — Additional file 5. Interview Guide (GP supervisors). [file 12913_2020_5684_MOESM5_ESM.docx]

**Interview Guide (GP supervisors)**

Participant ID: __________________________

Community Location: ____________________

Date of the Interview: ____/____/____

Interview Mode:

☐ In Person

☐ Over the Phone

Key Informants’ Gender:

☐ Male

☐ Female

The interview opens with:

*Hello, my name is* ***XXX****. I will be interviewing you today. The purpose of this interview is to understand the impact of JCU GP training registrars in rural and remote areas. JCU has been providing GP training across North West Queensland for the past 3 years building on the undergraduate medical training program and we are trying to better understand the effects of the program on registrars and communities. I will be audio recording this interview, which will be de-identified and remain confidential. Taking part in this study is voluntary and you can stop at any time without explanation or prejudice. Do you consent to participate in this interview?*

After consent is given, proceed to ask some demographic questions.

*Thank you for consenting to participate in this interview!*

*Before we start, I would like to know a bit about you.*

**Demographic Questions**

1. Could you please tell me your age range?

☐ 20 – 30

☐ 31 – 40

☐ 41 – 50

☐ 51 – 60

☐ 61 – 70

☐ 71 – 80

☐ Prefer not to say

2. How long have you lived in this community (in years)?

*Thank you very much for answering these questions.*

*Now I will ask you a few questions related to practice and life in a rural/remote community*

**Rural and Remote Area Impact**

1. Based on your experience, what has been the changes to your community/town because of JCU training GP doctors there?

2. a. Based on your experience, compared to 10 years ago or since you arrived, what differences have there been because of the establishment of JCU and the training of doctors in the region through JCU?

b. Can you describe or give examples of how GP training has impacted health service delivery?

3. What, if any of these impacts are directly related to JCU undergraduate medical training or GP training?

*If JCU is not involved, ask questions about the other universities helping in the GP training in the area and how they have helped the process? Or if any other university is helping along JCU, ask how ?*

4. What have been the effects of establishing GP training on specific populations in your community?

a. Aboriginal and Torres Strait Islander

b. Other populations such as farming, mining. *Question will depend on the demographic data of the area.*

5. What has been the impact of GP training and establishing local nodes on

a. The availability of healthcare services?

*Probing questions – examples – types of clinical care available*

*Health education/promotion*

*waiting times for opportunities*

b. Quality of medical services?

*Probing Questions – examples – being able to see the same doctor over time, feeling like doctor is listening to you, any improvement in your health or medical care*

*Can you elaborate with examples?*

c. The need to travel elsewhere for medical care?

d. Using telehealth services?

e. Have there been any examples that you can think of where the JCU GP training program has contributed to the local economy?

*Probing questions – hiring people from community, funding for education or health services, renting offices*

*Can you elaborate with examples?*

g. How has the commencement of JCU GP training program impacted or had any effect on the wider community?

*Are there any unintended consequences of this program on the community? Can you elaborate with examples?*

h. How have the JCU GP doctors or their families contributed to the community outside of work*? Can you please elaborate with examples/ or give examples?*

6. Have there been any changes to the recruitment of doctors and other healthcare providers as a result of the JCU undergraduate medical program or GP training? *Probing question – how? Can you please give examples?*

7. How would you describe the effect of establishing GP training in your community on the relationship between the community and

a. Post-secondary educational institutions (University and Colleges)

b. Health care service in your community or health service region

8. How has the community contributed or influenced the JCU medical school or GP training program?

9. What changes have been there in staff turnover or locum use since the introduction of GP training?

10. Are there any areas currently with no GP supervisors that you think would benefit from the establishment of GP training? *Can you please name few?*

11. Do you have any suggestions for attracting GP supervisors or engaging GPs to become supervisors in areas? *Can you please give examples?*

12. What would motivate a GP to become a supervisor?

b. What would deter a GP from becoming a supervisor?
