# Supplementary material for: How does GP training impact rural and remote underserved communities? Exploring community and professional perceptions
Source: BMC Health Serv Res. 2020 Aug 31;20:812. doi: 10.1186/s12913-020-05684-7 (PMC7457499; doi:10.1186/s12913-020-05684-7)
Supplement: Supplementary file 6 — Additional file 6. [file 12913_2020_5684_MOESM6_ESM.docx]

| **Reviewers’ Comments** | **Response** | **Page Number** |
| --- | --- | --- |
| **Reviewer 1** | | |
| I still think the economic contribution part presented under 'Financial Considerations' heading sits oddly with the rest of the qualitative paper - I still don't think it adds much. | The economic information was condensed and reworded to fit better with the background of the article. | Background, pg. 6, para 1, lines 6-13 |
| The aim of the study should not be in this section but much earlier on in the paper. | The Methods title before the aim was presented was a typo. The Methods title has been removed. | Background, pg.8, para 1, line 1 |
| The Methods section is still problematic. What were the characteristics of the 'purposive' sample? | The characteristics of the purposive sample were provided within the Participants section. Specifically, it is stated that “…previously identified areas of low medical workforce in Queensland were recruited for this study” and “Prospective participants were eligible to participate as long as they were residents of the targeted communities and had some knowledge of the training program.” Additionally, the relevant demographics of the participants are also presented in the Participant sections. The characteristics of the targeted population and the participants are identified within the article and as such, no changes were made. | - |
| The authors state that 'No limit was placed on the number of participants required as strict guidelines regarding sample sizes are not used in qualitative research due to the importance that is placed upon the individual's experience. A total of 40 participants took part in the project.'  It is true that there are no prescribed sample sizes in qualitative research - sizes tend to vary according to the nature of the topic, the heterogeneity of the population etc.  However, in this study there is no information about whether the 40 participants represented a good spread of the characteristics the purposive sample intended to capture.  The authors say that 'Recruitment and interviews continued until data saturation was reached.'  It isn't enough to announce that saturation has been reached with no more detail.  Better to say something like 'constant comparison between fresh data and earlier coded transcripts indicated that saturation was reached after x interviews and no new themes emerged after this point.' | Further detail regarding data saturation was included within the Procedure section. | Procedure, pg. 11, para 1, lines 4-7 |
| Also, as pointed out first time around, no information is given about how many people were contacted for interview and were any reasons given/sought for non-participation. | This point was addressed previously. As noted within the Participants section “Invitations to participate were sent by email through local networks to key informants in the underserved communities (such as training staff), relevant federal, state and local level government members, healthcare services and groups, and community volunteer groups (such as Rotary, Country Women’s Association).” As contacts were asked to pass on the project information through their networks, the exact number of people contacted cannot be identified.  No rationale is needed for non-participation in terms of people being able to provide informed consent without coercion or prejudice. Anyone who wanted to participate was interviewed. Based on these reasons, no changes were made. | - |
| Also, still no explanation of decision re interviews/vs the one focus group. | This point was addressed previously and further detail had been added. It is stated in the Procedure section that “The focus group was held with six registrars during a training day for ease of participation.” Based on this point, no changes were made. | - |
| This section is a bit thin - mostly a repeat of the results. | The Discussion section was revised to provide stronger interpretations of the results. | Discussion, pg. 18-20 |
| The authors state that 'as purposive sampling was used, participants were chosen non-randomly. Although this was done to gain a representative sample of the impact that GP training can have on a community, it does mean that the results and conclusions have to be interpreted with caution.'  However, purposive sampling is not undertaken to gain a representative sample - that's what random quantitative surveys tend to do.  With a purposive sample, you are attempting to find participants who possess characteristics that you are particularly interested in and that you consider will have an important bearing on the research, but you aren't going to achieve a representative sample that way.  I think this sentence indicates a misunderstanding of qualitative methods and also seems to imply that a large random sample would perhaps have been preferable. | A purposive sample was used to recruit individuals that fit the characteristics identified within the Participants section. The use of representative in the identified sentence was used to mean 'reflective' and not statistical representativeness. The limitations section was revised to avoid confusion. | Limitations, pg, 20-21 |
| **Reviewer 2** | | |
| "Australian Governments" in the first sentence of the abstract and introduction is awkwardly worded. I believe the authors are referring to government at multiple levels, but this could be clearer. Perhaps, "Substantial governmental funding in Australia has been invested to support the training…" or something similar in the abstract and additional revision in the introduction. | These two sentences were reworded. | Abstract and Background, pg. 2-3 |
| The abstract should mention the focus group. | The focus group was included in the abstract. | Abstract, pg. 2, para 2, line 13-14 |
| The authors use "healthcare" and "health care" inconsistently. Personally, I prefer two words. | Health care when a noun, healthcare when an adjective. All uses of both forms were reviewed within the article. | Whole of article |
