# Supplementary material for: How does GP training impact rural and remote underserved communities? Exploring community and professional perceptions
Source: BMC Health Serv Res. 2020 Aug 31;20:812. doi: 10.1186/s12913-020-05684-7 (PMC7457499; doi:10.1186/s12913-020-05684-7)
Supplement: Supplementary file 7 — Additional file 7. [file 12913_2020_5684_MOESM7_ESM.docx]

| **Reviewers’ Comments** | **Response** | **Page Number** |
| --- | --- | --- |
| **Reviewer 1** | | |
| Page 3, line 24-48 requires references for the information discussed. Lack of references | This paragraph was edited and additional references have been included. | 5-7 |
| Page 5, line 36 missing full stop after reference (14, 38 ) | A full stop was added. | 10 |
| **Reviewer 2** | | |
| Major:  Please add all information missing that should be provided using the COREQ checklist: http://cdn.elsevier.com/promis_misc/ISSM_COREQ_Checklist.pdf | COREQ Checklist and relevant information was included. | - |
| Abstract:  N and some sociodemographic data is missing. | Further information about the participants was added to the abstract. | 2 |
| Background:  How long (minutes?, miles?) have rural and remote community residents to travel to get access to healthcare?  How fast (days/weeks?) do they get an appointment with a GP/ with a specialist? | The length of travel to access healthcare would vary on the community and where the individual may live within that community. The background section provides a broad and general overview and context of the situation. The Method identifies the classification of remoteness for each of the communities from which participants were recruited. Based on these reasons, no changes were made. | - |
| Please provide more information on the "collaborative partnerships with communities, values based selection criteria and community-engaged placements in underserved areas". | This paragraph was edited. Further information was included within the ‘Developing a medical workforce ‘pathway’’ section. | 7-9 |
| How undergraduate students get into the program (what profile is of special interest? If rural origin is one of them, how it is defined? In case: How long has a student lived in a rural are before being accepted?). | Further information regarding the program was included within the ‘Developing a medical workforce ‘pathway’’ section. | 7-9 |
| Is there any difference in the costs of becoming a doctor within the program? | No, further information regarding costs was included within the ‘Developing a medical workforce ‘pathway’’ section. | 8 |
| Regarding "Distributed community-engaged training is considered an important component of socially accountable programs through reciprocal relationships established between universities or training providers and the communities in which the training is based": how long is the training in rural (remote) areas (weeks, months?)? | Further information regarding the program was included within the ‘Developing a medical workforce ‘pathway’’ section. | 7-9 |
| Method  Information given: "A total of 40 participants took part in the project. Participants had lived in their communities ranging from a couple of months to 63 years (Median = 12 years)." As well as the information in Table 1, belongs into the result section. | Demographic information of participants can be presented in the method and therefore, no changes were made. | - |
| How many persons did you contact to recruit 40 participants? | Contacts were asked to pass on the project information through their networks. The exact number of people contacted cannot be identified and therefore, no changes were made. | - |
| Results  Information given here: "In the years 2016-2018, direct JCU GPT expenditure was over $49 million without taking into account the additional indirect impact of re-spending of this money in local communities. Hogenbirk et al (2) conducted a similar study in Northern Ontario and developed a population based multiplier. Applying this approach in this context suggests a potential total direct, indirect and induced economic effects of approximately $90 million dollars when the flow on economic effects of re-spending in communities are taken into account."  As well as economic data shown in Table 3 does not seem to be results but rather background information. Please put the information in the background section.  (Generally: To cite literature within the result section is very uncommon.) | The economic information was moved to the Background section to provide further context for the study. | 9-10 |
| Qualitative research is not really generalizable, it is rather a method for building hypothesis or to deepen the understanding of behavior. Therefore first sentences of the discussion section should be expressed with more caution and in the Limitation section generalization should be discussed a little more in detail. | The first couple of sentence in the discussion were reworded. Further detail was added to the limitations regarding generalisability. | 21-24 |
| Please add selection bias to the limitations. | Further detail was added to the limitations regarding selection bias. | 23 |
| **Reviewer 3** | | |
| Title: The title gave me a good idea of what the paper was about, except the professional as well as community perspective is included. The title just refers to community perceptions. | The word ‘professional’ was included in the title. | 1 |
| Abstract: The abstract summarises the paper well but does not give a theoretical framework. | Within the abstract it is stated that:  Australian Governments have invested substantial funding to support the training of General Practitioners (GPs) to serve rural communities. However, there is little data on the impact of this expansion of training on smaller communities, particularly for smaller rural and more remote communities. Improved understanding of the impact of training on underserved communities will assist in addressing this gap and inform ongoing investment by governments and communities.  No changes were made. | - |
| No evidence is provided regarding the quality of the GP training and supervision so I recommend removing the words 'high quality' from the beginning of the last paragraph. | The words ‘high quality’ were removed from the sentence. | 2 |
| Introduction  'Distributed model' of education could need definition for international readers as could the size of 'small populations'. | Further explanation was provided for ‘distributed model’.  ‘Small populations’ varies across the communities. The Method identifies the classification of remoteness for each of the communities from which participants were recruited. No changes were made for this point. | 4 |
| Qualitative approach and research paradigm:  The introduction showed why the research needed to consider a range of outcomes. However, there was no subsequent link as to why a qualitative approach was appropriate. No theoretical framework or epistemological position was described. This matters because the authors also run the program they are describing and so there is a risk of confirmation bias. The impression is that the authors are more used to quantitative research and have applied its positivist approach to qualitative methods rather than explicitly thinking through and stating their epistemological position. | Further detail was added to the aim regarding why a qualitative approach was taken for this study. | 10 |
| Researcher characteristics and reflexivity: This is gap needs addressing. The authors have cited their associations but not how their roles in medical education could affect their conduct of the research. As employees of JCU, this needs to be explicit and how this interest in the program was handled when also studying it. | Further detail was added to the method regarding researcher characteristics. The main researcher conducting the interviews and focus group did not have a prior relationship with the training program nor the participants. | 13 |
| Sampling strategy: This is described as purposive but I think it is more of a convenience or interest sample. A purposive sample would target not just those who were interested but aims to include the full breadth of factors that might affect someone's experience of something. Factors that are known from the literature to affect experience of education and training in rural and remote areas should be included. In this instance sampling should explicitly include doctors whose families are living elsewhere and in community, and those whose partners can and cannot find work, those who trained in Australia or overseas, and those who selected rural and remote GP training and those who were only able to gain a place in GP training by agreeing to go rural. At present the reader is left wondering if the interviews were only held with registrars who were enjoying their placements and keen to be interviewed and to be loyal to JCU. Sampling should also seek out potential negative cases and this was not mentioned. | Purposive sampling is a sample based on characteristics of a population and the objective of the study. Convenience sampling differs from purposive sampling in that expert judgment is not used to select a representative sample of elements (Lavrakas, 2008). There were criteria involved in both the selection of communities, and in the methods targeted for recruitment. Additionally, anyone who wanted to participate that fit the criteria, was interviewed. Participants were not screened for whether they held positive or negative views before participating. Some further detail was added to the Participants section. | - |
| Likewise, how the community members were selected and their characteristics other than age and gender were not mentioned. | Anyone who wanted to participate that fit the criteria, was interviewed. Relevant demographic information was reported such as how long participants had lived in the community. Some further detail was added to the Participants section regarding recruitment. | 12 |
| Data collection methods: the paper would be strengthened by more information on the questions that were asked, including how these relate to the issues identified in the literature search. | Further information on the development of the interview guides and question exemplars have been added within the Procedure section. | 13 |
| Data collection instruments and technologies: as above, otherwise clear | Question exemplars have been added within the Procedure section. | 13 |
| Units of study: the gap in information regarding some characteristics known to impact on doctor's ability to thrive in rural areas are missing. E.g. location of primary training , family and partner location, rural upbringing, prior rural experience | The purpose of this study was to investigate the impact of GP training on the community rather than to investigate MDs’ experience of working in rural and remote settings. Based on this reason, no changes were made. | - |
| Techniques to enhance trustworthiness: nil. This is a big gap. There is no mention of reflexivity by the authors. There is no discussion regarding triangulation such as using audit trails or member checking . | Further detail was added to the Analysis section regarding the shared coding sessions and input from all authors. | 14 |
| Synthesis and interpretation: the results used a simple thematic analysis and were logically presented, but were quite a thin description of different categories with examples . | The purpose of this study was to provide an understanding of community perceptions towards the GP training that occurs in their community and therefore, thematic analysis was an appropriate choice for analysis. As a result, no changes were made. | - |
| I was not expecting to read about economic methods and results from the introduction, and I question the value of presenting this information. | The economic information was moved to the Background section to provide further context for the study. | 9-10 |
| Implications: this was covered but the methodological gaps in processes needed to produce trustworthy qualitative research means that the implications should be much more cautiously phrased | Wording was changed within the Discussion to more carefully phrase the implications. | 21-24 |
| Transferability: the results may be transferable to other situations but are again limited by the methodology | Further detail was added to the Limitations section regarding constraints to generalisability. | 23-24 |
| Contributions to the field: this makes some contribution but from a single viewpoint. A much stronger argument could be built if more data were collected from a wider range of participants, and across different jurisdictions . | The purpose of this study was to investigate community perceptions of the GP training offered within a particular region which may differ, for various reasons, to that offered elsewhere. Expanding the project to include a wider range of participants for different jurisdictions would dilute the main purpose of the project. Therefore, no changes were made. | - |
| **Reviewer 4** | | |
| Abstract: No mention of the quantitative aspects of the study. | Quantitative aspect was moved to the background to provide context of the study rather than be presented as part of the study. Therefore, no changes were made to the abstract based on this change. | - |
| Background: A quick explanation of GP training would be good - what exactly is a GP registrar? I.e. what stage in training ? | Further information was added to the Background regarding GP registrars. | 3 |
| I'm not sure I fully understood what localised GP training consists of - I think more explanation would be good on this point. | Further information was included to explain localised GP training. | 8 |
| Methods: The majority of my comments relate to this section, which I think needs more detail/explanation. How was the purposive sample selected? | Further detail was added to the Participants section regarding the criteria for selection. | 11-12 |
| What were the criteria? | Further detail was added to the Participants section regarding the criteria for selection. | 11-12 |
| Are 'community members' patients? This isn't explained. | Community members were not patients, they were general community member. The word ‘general’ was added to the first sentence of the Participants section. | 11 |
| How were participants recruited - by telephone, email, face to face? Recruitment strategy is not well described - 'key informants' and 'local networks' were used, but no detail about who/what they are. | Further detail was added to the Participants section regarding recruitment. | 11-12 |
| A total of 40 volunteers took part - out of how many who were contacted ? How many were contacted from each of the groups (supervisor, registrar etc.). | Contacts were asked to pass on the project information through their networks. The exact number of people contacted cannot be identified and therefore, no changes were made. | - |
| Were reasons sought or given by those who did not want to participate? | If people did not want to participate then they did not have to. No rationale is needed for non-participation in terms of informed consent. Anyone who wanted to participate was interviewed. Therefore, no changes were made. | - |
| Semi-structured interviews were carried out plus one focus group. What was the rationale for this? Need to justify. FGs and interviews generate different types of data - why were interviews mostly chosen with one FG? How many took part in the FG as opposed to interviews? Was the FG mixed or just one type of participant (e.g. community members)? | Further detail was added to the Procedure section regarding the focus group. | 13 |
| How were the interview questions/FG topic guide developed? Summary of topics covered would be good. | Question exemplars have been added within the Procedure section. | 13 |
| Who carried out the interviews/FG? Would this have influenced participants (if so this needs to be mentioned in Limitations)? | Further detail was added to the procedure regarding who carried out the interviews. | 13 |
| Was the schedule piloted? | The interview questions were piloted with four medical students and two registrars and the questions modified slightly for clarity. | - |
| The text says that 'interviews were audio-recorded and transcribed verbatim to generate themes'. This implies that transcribing magically generates themes! I think this needs rewording. | The words ‘to generate themes’ was removed from the sentence. | 13 |
| How did the two researchers come to agree on a unified coding system? Does 'shared coding sessions' mean that they each coded transcripts and then met to agree on codes and resolve any coding differences? | Further detail was added to the Analysis section regarding the shared coding sessions. | 14 |
| Results: Many results are expressed in terms of 'participants reported ...' but it isn't clear if there were any differences between different groups of participants. For example, did community members express certain views more than supervisors or registrars etc.? I would have expected some differences to emerge? | No overt differences were noted between groups in terms of the training’s impact on community. Supervisors and registrars were able to provide more in-depth information on the training itself however, the focus of this article is the impact on community. Based on this reason, no changes were made. | - |
| The results suffer from a lack of explanation, in particular, of who the 'community members' are - as noted earlier. Are they a sample of patients? Or are they just random people from the community? | This issue was addressed above and further detail was added to the Participants section. | 11-12 |
| It is reported that 'participants noted that the localised GP training played an important role in the recruitment & retention of GPs.' I think there needs to be more hard evidence here - did the scheme result in GPs coming back to work in the community after training? Did it lead to posts being filled more easily? | The purpose of the study was to gain community perceptions of the impact that training had on the community. Although numbers around this point may be beneficial, it was beyond the scope of the current project. Therefore, no changes were made. | - |
| I think the economic impact section sits a little oddly within what is overwhelmingly a qualitative evaluation. I almost wondered if that should be left out. | Quantitative aspect was moved to the background to provide context of the study rather than be presented as part of the study. | 9-10 |
| Limitations: Data saturation should be discussed earlier on in methods section. At what point was data saturation reached? The impression given is that 40 people agreed to take part and so 40 were interviewed and then it was assumed that this would be enough. Forty is a good sample size for qualitative research, but how did the researchers come to the conclusion that saturation had been reached? Often, coding proceeds in tandum with data collection, so that researchers can see when no new codes are being added to the coding frame and can make an assessment about saturation. There needs to be more discussion about this. | Further detail regarding data saturation was added to the Procedure section. The Analysis section also outlines that the analysis occurred in an iterative process however, an additional sentence was added to clarify that analysis occurred while interviews were still being conducted. | 13-14 |
